# Prevalence and risk factors for diabetes mellitus among tuberculosis patients in Moshi Municipal Council, Kilimanjaro Tanzania

**DOI:** 10.24248/eahrj.v5i1.658

**Published:** 2021-06-11

**Authors:** Patrick L. Mabula, Kelly I. Kazinyingia, Edwin Christian Chavala, Victor Mosha, Sia E. Msuya, Beatrice John Leyaro

**Affiliations:** a Kilimanjaro Christian Medical University College (KCMUCo), Tanzania; b Department of Community Health, Institute of Public Health, Kilimanjaro Christian Medical University College, Moshi, Tanzania; c Department of Epidemiology and Biostatistics, Institute of Public Health, Kilimanjaro Christian Medical University College (KCMUCo), Tanzania; d Community Health Department, Kilimanjaro Christian Medical Centre (KCMC), Moshi, Tanzania; e Kilimanjaro Christian Medical Centre (KCMC), Moshi, Tanzania

## Abstract

**Background:**

Diabetes Mellitus (DM) is a worldwide public health problem and its prevalence has been rising rapidly in low and middle income countries (LMICs) including Tanzania. According to WHO report 2015, DM is ranked number six as a leading cause of death worldwide. Strong evidence suggests that DM may be associated with Tuberculosis (TB) and could affect TB treatment outcomes. Tanzania is among the 22 countries that have a high burden of TB and currently facing increased epidemic of DM. The increasing diabetes prevalence may be a threat to TB control and counteract strategies to end TB by 2030 as proposed by WHO.

**Objective:**

To determine proportion of TB patients who are co-infected with DM in Moshi municipal council, Kilimanjaro Tanzania.

**Methodology:**

This study was a hospital based cross-sectional study conducted in April to July 2018 at 4 health facilities; Mawenzi Regional Referral hospital, St. Joseph District Designated hospital, Pasua Health center and Majengo Health centre in Moshi municipal. The study included adults aged 18 years and above attending either of the 4 health facilities for TB care. The study included newly diagnosed and those who were on TB treatment. Interviews were conducted followed by blood glucose testing. Data was entered and analysed using SPSS

**Results:**

A total of 153 TB patients were enrolled, their mean age was 42.5 (±14.75) years and 46 (30.1%) were females. The prevalence of DM among TB patients in this study was 9.2%. Factors associated with TB-DM comorbidity were: age (OR 4.43, 95% CI: 1.18–16.55), HIV status (OR 3.88, 95% CI: 1.06–14.11), and family history of DM (OR 6.50, 95% CI 0.67–25.56).

**Conclusion:**

One in ten patients with TB had confirmed DM. There is a need for future studies to assess if DM influences TB treatment and outcomes in this setting.

## BACKGROUND

Tuberculosis (TB) is an infectious disease caused by bacteria (mycobacterium tuberculosis) that oftenly affects the lungs. Tuberculosis is spread from person to person through the air. When people with pulmonary TB cough, sneeze or spit, they propel the TB germs into the air.^1^ Tuberculosis and Diabetes Mellitus are major causative of mortality across the world.^2^ TB is one of the top 10 causes of death globally. It is estimated that 9.0–11.1 million people developed TB in 2017 and approximately 1.3 million died.^3^ Tuberculosis and Diabetes Mellitus (TB-DM) comorbidity is higher as compared to TB-HIV co-infection around the world. It is estimated that in 9.6 million new cases of active TB annually, 1 million have both TB and DM.^4^ With more than 70,000 cases each year, Tanzania is among the 22 high burdened TB countries where by the dual burden of TB and DM has become a major health threat^5–7^

Diabetes Mellitus increase the risk of TB by threefold^8–10^; it impairs innate and adaptive responses that are necessary to counter the progression from infectious to clinical disease. DM is an independent risk factor which cause poor TB treatment response as well as death.^11,12^ The relationship between DM and TB is assisted by the fact that patients with DM have impaired cell mediated immunity, renal failure, micronutrient deficiency, and pulmonary microangiopathy, all of which increases the susceptibility of *myco-bacterium tuberculosis*.^13^

The dual burden of TB and DM in both low and midd le-income countries has become a global health prob-problem. Several studies have suggested that there is an association between DM and TB and the possible interlink represent an important and growing challenge of TB global control.^14^ Conversely, Active TB patients experience Inflammation caused by cytokines such as IL6 and TNFa in response to TB infection which may cause an increase in insulin resistance and decreased insulin production, thereby leading to hyperglycemia.^15^ Additionally, isoniazid and rifampin have hyperglycaemic effects. Also pyrazinamide may result in difficult control of DM. Rifampin induces metabolism and decreases blood level of sulfonylureas, leading to hyperglycemia.^15^

In recognition of the burden of DM and TB, the World Health Organization (WHO) and the International Union against Tuberculosis and Lung Disease (The Union), launched the Collaborative Framework for Care and Control of Tuberculosis and Diabetes to guide policy makers and programme managers in combating the TB-DM epidemic.^16^ However, recent studies have shown that DM prevalence among TB cases is variable and it ranges from 29.3% in Southern Mexico to 11.4% in Georgia USA^17,18^ Studies conducted in Africa reported low prevalence of DM among TB patients; 8.5% in Uganda, 8.3% in Ethiopia, 2.8% in Guinea-Bissau and 1.9% in Benin.^13,19–21^ Some studies have shown high prevalence of DM among TB patients; 29.5% in Taiwan, and 25.3% in India^22,23^

In Tanzania, the comorbidity of DM and TB has not been of great concern as a growing health threat at the national level. Although, National Tuberculosis and Leprosy Programme identified 2 major risks of TB; one being HIV for which patients have an annual risk of 5-10% of developing TB, DM which has an annual of 1.5 times higher risk of developing TB.^24^ Therefore, DM is increasing among TB patients making it one of the risk burdens of TB in Tanzania. Considering that two thirds of DM patients in Africa are not aware that they have the disease, screening for DM among TB patient may contribute to early diagnosis.^16^ However, there is no recommendation from the ministry of health and social welfare to all health facilities about DM screening to new TB patients as compared to the screening for HIV. In Tanzania, there is limited information on DM among TB patients, therefore this study wants to determine Prevalence and factors associated with DM among TB patients in Moshi Municipal council. Information from this study may contribute to the Community, Ministry of Health and social welfare, and National Tuberculosis and leprosy Programme, the need for routine screening of DM among TB patients and appropriate strategies for interventions.

## METHODOLOGY

### Study Design

A cross-sectional study was conducted among TB patients attending health facilities that offer TB treatment in Moshi Municipality, Tanzania from April to June 2017.

### Study Population

All TB patient aged 18 years and above attending selected TB health facilities at Moshi Municipality in Kilimanjaro Region. Patients who were not permanent residents of Moshi Municipality were excluded from the study.

### Study Area

Kilimanjaro region is located in North-eastern part of Tanzania mainland. It has 7 districts including Moshi municipal council.

This study was conducted in 4 Health facilities offering TB services in Moshi Municipal council namely; Mawenzi regional referral hospital, St joseph hospital, Majengo hospital, and Pasua health Centre.

### Sample Size Estimation

Sample size (N) was determined by using a precision formula $\text{N}={\text{Z}}^{2}\text{p}(1-\text{p})/{\text{e}}^{2}$,

whereby **Z** is Standard Normal Deviation of 1.96 corresponding to 95% Confidence Interval, **P** is Population prevalence of 16.7% from a Descriptive Cross Sectional study titled **Diabetes is a risk factor for pulmonary tuberculosis, a case control study from Mwanza Tanzania**, and e is Precision set at 5% (0.05).

The required minimum sample size of 213 was obtained from the above estimation and the final sample size was determined to be 234 TB patients after additional of 10% for non-response rate.

### Sampling Technique

All TB patients aged 18 years and above who visited the selected health centres for clinic during the study period were enrolled.

### Study procedures

We collected Information concerning the socio-demographic and the associated factors using a pre-tested standard questionnaire. After patient agreement, he/she filled the questionnaire for collection of socio-demographic factors and risk factors of DM among TB patients on their visit for commencement of TB treatment. DM was investigated as follows: Random blood glucose (RBG) test was performed, if the level is less than 11mmol/L, no further action was taken. If RBG is more than 11mmol/L, then the patient was asked to return for Fasting Blood Glucose (FBG) the next morning. A glucometer ACCU-CHEK Active Glucose Monitoring System (Roche Diabetes care Inc. Indian) was used for screening DM. WHO diagnostic criteria were used for making a diagnosis of DM, i.e. Fasting Blood Glucose (FBG) value of ≥ 8mmol/L. Patients found to be diabetic were referred to the diabetes clinic of their respective facility for further evaluation and management. Moreover, known DM patients were educated on the need to continue follow-up care for DM.

### Data Collection Tools

Questionnaires in Swahili language were used for data collection. The questionnaire had 2 sections; section one included socio-demographic information such as age, education status and marital status. Section two included information on risk factors for DM such as HIV status, alcohol consumption, cigarette smoking and participants’ family history of DM. Smoking was categorised as ever smoke or never smoke in life time, alcohol was categorised as ever drink or never drink in a life time and family history of DM involved asking if there is a first degree relative with DM, information about HIV status of the newly diagnosed and under medication participants were retrospectively obtained from the TB registry book in every clinic, DM was measured using a glucometer ACCU-CH-EK Active Glucose Monitoring System (Roche Diabetes care Inc. Indian) A blood sample was collected through a finger prick and dropped on a glucometer to give a reading. Information concerning socio demographic and associated factors was collected by using a pre-tested standard questionnaire administered by trained research assistants.

### Data Collection Methods and Tool

Data was collected by conducting interviews guided by Questionnaires. The questionnaires contain information on social demographics, information about risk factors associated with DM among TB patients, microangiopathy etc Biological samples collection tools were used for collection of samples for DM screening among TB patients.

### Laboratory Procedures and Machine

A glucometer machine (ACCU-CHEK Active Glucose Monitoring System (Roche Diabetes care Inc. Indian) uses strips. The strips contain chemicals that react with glucose in the drop of approximately 0.3 to 1μl of blood. It takes about 3 to 60 seconds to read the test strip; the glucose value in mg/dl is displayed on a digital display. Laboratory procedures were practiced as follows; Patients’ fingertips were cleaned using 70% alcohol swabs. A new glucometer strip was inserted for each test for every individual patient. A spot on a cleaned patient's finger was chosen and patient's fingertip was lanced to get a drop of blood. The test strip was held close to the lanced fingertip to collect the blood drops until enough blood is absorbed to begin the test. It takes about 3 to 60 seconds to read the test strip. The glucose value in mg/dl is display on a digital display. Used test strips and lancets were discarded properly, the results were recorded on the patients’ request forms.

Patients were given their results and those with diabetes were referred to the diabetic clinics in their respective health centres.

### Data Quality

During data collection, a daily basis check-up of the questionnaires and patient request forms was done to ensure the correctness and completeness of the information collected.

### Data Analysis

Statistical Package for Social Sciences (SPSS) version 20 (SPSS, Chicago, IL) was used for data analysis. Descriptive analysis was done for data summarisation, whereby mean and its corresponding measures of dispersion were used for numeric variables, frequency and percentages for categorical variables. Odds Ratio (OR) and the corresponding 95% Confidence Interval (CI) was used to determine factors associated with DM using logistic regression models. All variables which were statistically significant at the univariate analysis were carried for multivariable analysis. A p-value of <0.05 was considered statistically significant.

### Ethical Consideration

Ethical approval to conduct the study was obtained from the Kilimanjaro Christian Medical University College Ethical Committee (Ethical approval number 2039). Permission to carry out the study was obtained from the District Medical Officer (DMO) of Moshi Municipality and in-charge of selected health facilities. Written, informed consent was obtained from each participant. Confidentiality with regards to the information of the participants was highly maintained as no names appeared on the questionnaires. Participants who did not want to participate were not denied access to services at the health facilities. Participants with raise FBG of 7 mmol/L were referred to the diabetes clinic of their respective facility for further evaluation and management.

## RESULTS

### Socio-Demographic Characteristics of the Study Participants

A total of 153 participants were analysed in this study. The overall mean age of the participants was 42.5years (standard deviation (SD) (±14.75). Majority 107(69.9%) of the participants were male and about 77(51.6%) aged between 18-39 years. For other socio-demographic characteristics of the study participants, See Table 1.

**TABLE 1:** Socio-Demographic Characteristics of the Participants (N=153)

Variables	N	%
**Sex**		
Male	107	69.9
Female	46	30.1
**Age**		
18-39	79	51.6
> 40	74	48.4
**Educational level**		
Formal education	144	94.1
Informal education	9	5.9
**Marital status**		
Single	45	29.4
Married	108	70.6
**Occupation**		
Employed	27	17.6
Unemployed	39	25.5
Self employed	87	56.9

### Prevalence of Diabetes Mellitus among TB patients

The prevalence of DM in this study was 14(9.2%). The prevalence was 11(14.9%) higher among older age group compared to the younger group, also higher 4(23.5%) among HIV positive patients and patients with family history of DM.

### Factors Associated with Diabetes Mellitus among TB Patients

Table 2 shows univariable and multivariable analysis on the factors associated with DM among TB patients. The significant factors found to be associated with DM were age, family history of DM and HIV status. Participants aged 40 years and above had 4.42 times higher odds of developing DM as compared to younger age group (1839) (OR 4.4295% CI 1.18–16.55) and the association was statistically significant. Also, participants with family history of DM had 6.5-time higher odds of developing DM as compared to patients with no family history of DM (OR 6.5001, 95% CI 0.67–25.56), likewise participants who were HIV sero-positive had 3.8 times higher odds of developing DM as compared to those who were HIV-negative (OR 3.88, 95% 1.06–14.11).

**TABLE 2:** Factors Associated with Diabetes Mellitus among TB Patients (N=153)

Variables	Diabetes Mellitus			
	N	Diabetic n (%)	OR (95%CI)	AOR(95%CI)
**Sex**				
Male	107	12 (11.2)	2.77 (0.59–12.95)	2.91 (0.52–16.17)
Female	46	02 (4.3)	1	1
**Age**				
18–39	79	03(3.8)	1	1
≥40	74	11(14.9)	4.42 (1.18–16.55)	7.68 (1.38–42.72)
**History of smoking**				
Yes	67	09(13.4)	2.51 (0.80–7.89)	
No	86	05(5.8)	1	–
**Alcohol consumption**				
Yes	116	11(9.5)	1.18 (0.31–4.51)	
No	37	03(8.1)	1	–
**Family history of DM**				
Yes	12	04(33.3)	6.50 (1.68–25.56)	17.5 (2.64–116.07)
No	141	10(7.1)	1	1
**Physical exercise**				
Yes	46	05(10.9)	1.32 (0.42–4.21)	–
No	107	09(8.4)	1	
**HIV status**				
Positive	17	04(23.5)	3.87 (1.07–14.12)	2.5 (0.59–10.64)
Negative	136	10(7.4)	1	1
**Marital status**				
Single	45	01(2.2)	6.02 (0.76–47.88)	
Married	108	13(12.0)	1	–
**Education status**				
Formal education	144	13(9.0)	0.79 (0.09–6.85)	
Informal education	09	01(11.1)	1	–

## DISCUSSION

The prevalence of DM among TB patients in Moshi municipality was 9.2%. The factors significantly associated with TB-DM comorbidity were age (> 40), HIV status and family history of DM.

The prevalence of DM among TB patients in this study is comparable with that reported in Dar es salaam Tanzania, (9.7%), Nigeria (9.4%), and a pooled prevalence of a meta-analysis study of SSA (9.0%).^11,16^,^25^ However, the prevalence is lower compared to that reported in Pakistan (14.8%), India (13.9%) and Bangladesh (12.8)^26–28^ Other studies have reported lower prevalence as compared to our study; Uganda (8.5%), Tanzania (4.5) Lusaka Zambia (2.3%), Mozambique (1%).^19^-^29^-^30,31^ The possible explanations for the difference could be difference in study setting and screening method used in DM diagnosis, for example, in the study conducted in Pakistan^26^, both FBG and HBA1C were used for DM screening which is more effective as compared to the method used in the current study. Also, the present study was conducted on a relatively small sample size compared to the sample sizes considered in other studies.

The odds of DM was higher among participants aged 40 years and above and having family history of DM. This may be explained by the fact that type two diabetes is common in old age people compare to young age people and the possibility of inheriting DM traits. Our findings are similar to the study conducted in Dar es salaam Tanzania, Kenya and Ethiopia^11–32–33^, but contrary to that of Nigeria^34^ where no difference in the odds of DM was found.

HIV infection was a significant risk factor for TB and this is similar to the study done in Ethiopia.^33^ This can be explained by the fact that HIV infection weakens the immune system and increase the risk of TB, however, this contrary to the study done in Kenya.^32^

This study shows no association between sex, education level, marital status and occupation of the study participants and this is similar to a community based cohort study conducted in China and a cross sectional study conducted in Pakistan.^26,35^ However, this is contrary to a systematic review by Workneh *et al.* 2017 and a study conducted in Bangladesh.^28,36^ The possible explanation for this difference could be due to the difference in the study sample size. This study's sample size was small compared to other studies.

### Study Strength and Limitations

We conducted a DM screening programme among all TB patients across 4 health facilities in Moshi municipality. The study has the following limitations; utilisation of FBG for diagnosis. FBG has low sensitivity and may fail to detect DM in some patients which may have caused under estimation of the prevalence of DM. FBG is recommended as initial DM screening test in resource-limited settings; Using of A glucometer machine (ACCU-CHEK Active Glucose Monitoring System). The glucometer is less sensitive for diagnosis when compared to other diagnosis methods such as HBA1C; The Small sample size used in this study resulted into having a wide Confidence Interval, thus, precaution should be taken while interpreting the outcomes of this study.

## CONCLUSION

In this study, we found that at least one in ten patients with TB had confirmed DM. Therefore, we strongly recommend to routine screening for DM among all TB patient and special emphasis should be given for early screening of DM among TB patients so as to contribute to improved detection and early treatment.
